# Presynaptic depolarization differentially regulates dual neurotransmitter release from starburst amacrine cells in the mouse retina

**DOI:** 10.3389/fopht.2023.1225824

**Published:** 2023-08-29

**Authors:** Tomomi Ichinose, Chase B. Hellmer, Jeremy M. Bohl

**Affiliations:** Department of Ophthalmology, Visual and Anatomical Sciences, Wayne State University School of Medicine, Detroit, MI, United States

**Keywords:** acetylcholine, GABA, synapse, kinetic, postsynaptic currents, EPSCs, iPSCs, optogenetic

## Abstract

The retina is comprised of diverse neural networks, signaling from photoreceptors to ganglion cells to encode images. The synaptic connections between these retinal neurons are crucial points for information transfer; however, the input-output relations of many synapses are understudied. Starburst amacrine cells in the retina are known to contribute to retinal motion detection circuits, providing a unique window for understanding neural computations. We examined the dual transmitter release of GABA and acetylcholine from starburst amacrine cells by optogenetic activation of these cells, and conducted patch clamp recordings from postsynaptic ganglion cells to record excitatory and inhibitory postsynaptic currents (EPSCs and IPSCs). As starburst amacrine cells exhibit distinct kinetics in response to objects moving in a preferred or null direction, we mimicked their depolarization kinetics using optogenetic stimuli by varying slopes of the rising phase. The amplitudes of EPSCs and IPSCs in postsynaptic ganglion cells were reduced as the stimulus rising speed was prolonged. However, the sensitivity of postsynaptic currents to the stimulus slope differed. EPSC amplitudes were consistently reduced as the steepness of the rising phase fell. By contrast, IPSCs were less sensitive to the slope of the stimulus rise phase and maintained their amplitudes until the slope became shallow. These results indicate that distinct synaptic release mechanisms contribute to acetylcholine and GABA release from starburst amacrine cells, which could contribute to the ganglion cells’ direction selectivity.

## Introduction

Visual perception begins at the back of the eye in the retina, where many types of neurons form neural circuits. Photoreceptors encode photon flux reflecting from the environment and transmit this information to downstream neurons, including approximately fifteen types of bipolar cells, sixty types of amacrine cells, and dozens of ganglion cells (1–8). Unique synaptic connections between distinct types of neurons are thought to form multiple parallel neural circuits that process particular aspects of visual signals, such as color and motion (9–13).

Among retinal circuits for visual feature detection, one of the best characterized neural circuits enables tracking the direction of a moving object, including type 2, 5, and 7 bipolar cells (14–17), starburst amacrine cells (SACs) (9, 18), and direction-selective ganglion cells (19–21). SACs are neurons that demonstrate asymmetric direction selective responses to moving objects, and are comprised of “ON” and “OFF” types that track bright or dark moving edges (22, 23). Furthermore, SACs exhibit larger “preferred” directional responses for centrifugal motion (from soma to peripheral dendrites) than for “null” directional responses for centripetal motion (from peripheral dendrites to the soma). Ca^2+^ imaging from the SAC distal dendrites reveals a directional response with significantly higher Ca^2+^ signals for preferred rather than null moving stimuli (18, 24, 25), suggesting that objects moving in the preferred direction facilitate greater neurotransmitter release from SAC peripheral dendrites. In contrast, voltage responses from the SAC soma by patch clamp recordings exhibit directional responses in a different manner. In addition to showing subtle amplitude differences, a moving stimulus in the preferred direction evokes a steeper depolarization response than motion in the null-direction (24, 26, 27). However, the significance of the distinct depolarization kinetics in motion detection circuits has not been understood.

To address these questions, we used a mouse line with channelrhodopsin2 (ChR2)-expressing SACs and conducted patch clamp recordings from postsynaptic retinal ganglion cells (RGCs). We stimulated the ChR2-expressing SACs with bright light that varied in stimulus rise time while recording excitatory and inhibitory postsynaptic currents (EPSCs and IPSCs) from RGCs. We found that distinct SAC depolarization kinetics differentially evoked postsynaptic excitation and inhibition, which may contribute to the asymmetric directional responses of downstream motion-sensing RGCs.

## Methods

### Animals

Experiments were performed using healthy adult mice (4-12 weeks old, male or female). The Chat-IRES-Cre mice (RRID : IMSR_JAX:031661) were crossed with Ai32-ChR2-YFP mice (RRID : IMSR_JAX:024109) for optogenetic experiments. Animals were housed in 12-hour light-dark cycles. All animal procedures were approved by the Institutional Animal Care and Use Committee at Wayne State University (protocol no. 17-11-0399). All the necessary steps were taken to minimize animal suffering. The tissues were harvested immediately after the animal was euthanized by CO_2_ inhalation and cervical dislocation.

### Retinal preparation

The experimental techniques were similar to previously described (6, 16). Briefly, mice were dark-adapted at least one hour prior to dissection. The eyes were enucleated and the retina was isolated and cut into flat-mount preparations. All procedures were performed in dark-adapted conditions under infrared illumination using infrared viewers. The dissecting medium was cooled and continuously oxygenated. Retinal preparations were stored in an oxygenated dark box at room temperature.

### Whole-cell recordings

Whole-cell patch clamp recordings were made from YFP labeled ON-SACs or blindly targeted RGC somas in wholemount retinal preparations by viewing them with an upright microscope (Slicescope Pro 2000, Scientifica, UK) equipped with a CCD camera (Retiga-2000R, Q-Imaging, Surrey, Canada). Tissues were immobilized using a platinum horseshoe net with nylon wires over the tissue. L-EPSCs and L-IPSCs were recorded from ganglion cells by voltage clamping held the membrane potential at -55 and 0 mV, respectively. Light-evoked voltage responses were recorded from SACs at the resting membrane potential. All recordings were performed at 32-34°C. The electrodes were pulled from borosilicate glass (1B150F-4; WPI, Sarasota, FL) with a P1000 Puller (Sutter Instruments, Novato, CA) and had resistances of 6–9 MΩ. Clampex and MultiClamp 700B (Molecular Devices, San Jose, CA) were used to generate the waveforms, acquire the data, and control light stimuli by a light-emitting diode (LED) (Cool LED, Andover, UK). The data were digitized and stored on a personal computer using Axon Digidata 1440A (Molecular Devices). The responses were filtered at 1 kHz with the four-pole Bessel filter on the MultiClamp 700B and sampled at 2–5 kHz.

### Solutions and drugs

The retinal dissections were performed in HEPES-buffered extracellular solution containing the following (in mM):115 NaCl, 2.5 KCl, 2.5 CaCl_2_, 1.0 MgCl_2_,10 HEPES, and 28 glucose, adjusted to pH 7.37 with NaOH. Physiological recordings were performed in Ames’ medium buffered with NaHCO_3_ (Millipore-Sigma) and bubbled with 95% O_2_ and 5% CO_2_; the pH was 7.4 at 32 – 34°C. The intracellular solution contained the following (in mM):110 potassium methylsulfonate, 10 HEPES, 4 EGTA, 5 NaCl, 5 KCl, 1 MgCl_2_, 4 ATP-Mg, and 1 GTP-Na, adjusted to pH 7.2 with KOH. For voltage-clamp recordings, the intracellular solution contained the following: 110 cesium methylsulfonate, 10 HEPES, 10 TEA-Cl, 4 EGTA, 1 MgCl_2_, 5 mM QX-314, 4 ATP-Mg, and 1 GTP-Na, adjusted to pH 7.2 with CsOH. To block photoreceptor inputs to bipolar cells and SACs, 10 µM L-AP4 (Tocris), 1 µM ACET (Tocris), and 50 µM GYKI53655 (Tocris, Bristol, UK) were perfused in the bath solution.

### Optogenetic stimulation

Retinal wholemount tissues were light-adapted at 1 x 10^5^ photons/µm^2^/s in the recording chamber. Photoreceptor blockers, described above, were bath applied to isolate ChR2 evoked current. A 1s step light (500nm, 1 x 10^10^ photons/µm^2^/s, 150µm diameter) was used to optogenetically depolarize ON and OFF-SACs. Then, the rising phase of the stimulus was altered from 10 to 990 ms to reach the peak stimulus amplitude, while the total stimulus duration was held to 1s for all stimuli. Optogenetic stimuli had interstimulus intervals of 10s.

### Data analysis

SAC depolarization phase was curve fit with a double exponential equation:


$$f\left(t\right)=\sum_{i=1}^{n}Ai*{\left(1-e^{-t/\tau i}\;\right)}^{a}+C$$

For SAC depolarization analysis, a mixed-model repeated-measures ANOVA was used. For RGC EPSCs and IPSCs analysis, a mixed-model ANOVA was used to compare the amplitudes between 10ms and other time-evoked responses (Prism v.9, GraphPad Software, San Diego, CA). The repeated and mixed-model ANOVA were run with a Geisser-Greenhouse correction to account for possible violations of the assumption of circularity/sphericity, followed by a Tukey’s multiple comparisons test to obtain the adjusted *p-value*s.

## Results

**Figure 1:**
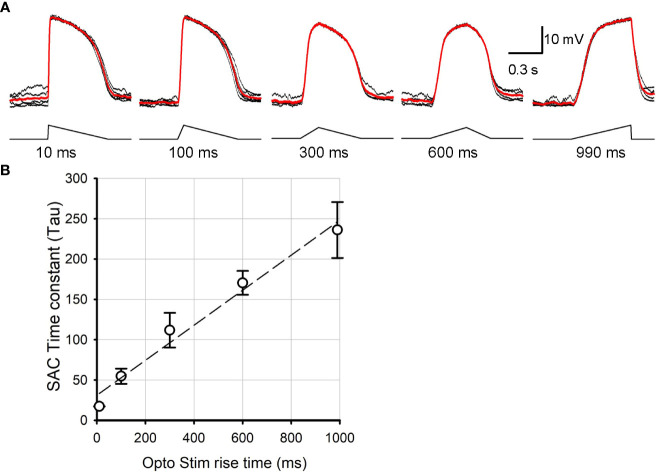
SAC voltage changes in response to distinct kinetics of ontogenetically stimuli. **(A)** SAC voltage responses evoked by 10 ms, 100 ms, 300 ms, 600 ms, and 990 ms triangle rise time stimuli. Traces in black were individual sweeps, and the trace in red indicate an average of five recordings. The rise time is indicated below each stimulus. **(B)** A summarized graph showing the time constant (tau) of SAC response rise phase as a function of the stimulus rise time (N=5 SACs). It revealed that ChR2 responded linearly as the light stimuli temporal aspects changed.

The ChR2-expressing SAC mouse line (Ai32 x ChAT-cre) has been used to study SAC synaptic transmission (16, 28, 29). We first examined the kinetics of ChR2-mediated depolarization of SACs in response to varied optogenetic stimulus rise times, mimicking the steep vs. shallow rise times of the asymmetric depolarization evoked by preferred vs. null directional stimuli (24, 26, 27). We conducted whole cell patch clamp recordings from ON SACs in SAC-ChR2 mice while pharmacologically isolating them from photoreceptor input. SAC cell identity was confirmed by observing their unique morphology using YFP fluorescence and IV relations (16, 22). ChR2-mediated depolarization of SACs was evoked in response to optogenetic stimuli of 10, 100, 300, 600, and 990 ms rise times (**Figure 1A**). SACs depolarized with distinct kinetics to the optogenetic stimuli with varied rise time. Because the depolarization phase has non-linear kinetics, we fit an exponential curve (see Method section) and compared the time constant (tau) for the initial depolarization phase in response to distinct optogenetic stimuli (**Figure 1B**). The correlation of the curve fit exhibited above 98% for all cases. The time constant increased when the rise time of optogenetic stimulus increased (p<0.05, N=5 SACs, repeated ANOVA). The result indicated that the ChR2-SAC mouse line is suitable for investigating how subtle temporal changes in SAC depolarization evokes differential postsynaptic excitation and inhibition.

**Figure 2:**
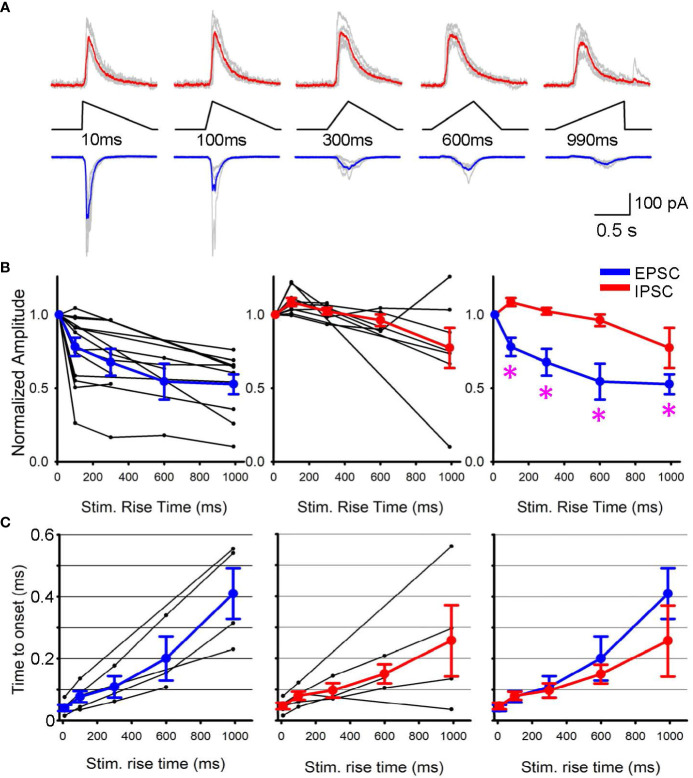
EPSPs and IPSPs varied when the rising phase of SAC stimuli changed. **(A)** Representative Sweeps of IPSCs (upper) and EPSCs (lower) obtained from a ganglion cell in response to optogenetic stimuli of 10 ms to 990 ms rise times. **(B)** (left) Normalized EPSCs in ganglion cells as a function of SAC stimuli rise time (black, n=15 RGCs) with average responses shown in blue. (middle) Normalized IPSPs (black, n=9 RGCs) with average IPSC shown in red. (right) The average EPSCs and IPSCs amplitudes are plotted with asterisks displaying a p< 0.05 when comparing the IPSC and EPSC response of the same triangle rise time. **(C)** The latency between stimulus onset to response onset time. Five RGCs that exhibited both EPSCs and IPSCs were selected and compared their latencies. (left) EPSC latencies and average in blue. (middle) IPSC latencies and average in red. (right) The average EPSC and IPSC latencies were overlaid, displaying no differences.

Subsequently, we conducted patch clamp recordings from postsynaptic RGCs to assess the outcome of differential SAC depolarization kinetics. We initially applied a step light optogenetic stimulus, and if the cell exhibited ChR2-evoked postsynaptic currents, we proceeded with further recordings. Because recordings were conducted in the presence of glutamate receptor blockers to isolate ChR2 activation (detailed in the Method section), EPSCs and IPSCs in RGCs were considered cholinergic and GABAergic, respectively (**Figure 2A**). When we prolonged the rise time of the optogenetic stimulus, EPSC amplitude decreased (**Figure 2B**, left). On average, EPSCs decreased significantly when the stimulus rise time increased (p<0.05, repeated ANOVA, n=15 RGCs from 10 mice, each time point consists of 4-15 cells, **Figure 2B**, left).

In contrast, IPSCs were less sensitive to changes in stimulus rise time. Although the stimulus rising phase was prolonged, IPSC amplitudes were similar up to 300 ms (p>0.1, repeated ANOVA, n=9 RGCs from 7 mice, each time point consists of 4-9 cells, **Figure 2B**, middle). The average EPSC and IPSC amplitudes were overlaid in **Figure 2B** right, showing that EPSC amplitudes were more susceptible to the rising phase of SAC depolarization. In contrast, the latency between the stimulus and response onset did not show a difference between EPSCs and IPSCs (**Figure 2C**). These results suggest differential release of GABA and acetylcholine at SAC-RGC synapses. The differential synaptic transmission of excitation and inhibition could contribute to generating direction selective signaling in postsynaptic RGCs.

## Discussion

SACs possess a unique and exquisite dendritic morphology, which has been investigated for decades, leading to their identification as a crucial component of retinal motion detection (9, 18, 30). SACs also contain dual neurotransmitters, releasing both GABA and acetylcholine, similar to dozens of other types of amacrine cells that release multiple neurotransmitter types, however SACs are the only type of amacrine cells to release acetylcholine. (8). Even with this unique transmitter combination the synaptic release mechanisms of SACs’ are not fully understood.

Lee et al. (31) conducted dual patch clamp recording from SACs and postsynaptic direction-selective ganglion cells (DSGCs), and found that DSGCs receive both GABAergic and cholinergic synaptic transmission from SACs. However, the Ca^2+^-dependency of SAC transmitter release differs; cholinergic release requires high Ca^2+^ in the intracellular solution (1.5 mEq), whereas GABAergic release occurs even with low Ca^2+^ in the intracellular solution (0.2 mEq). They further found that differential Ca^2+^ channels regulate the dual transmitter release: N-type for cholinergic and P/Q-type for GABAergic transmission. The P/Q channels and N channels exhibit slightly different voltage gated dependencies (32, 33), which may generate differential synaptic transmission.

Differential transmission from SACs has been reported. Pottackal et al. (29) used optogenetic SAC stimulation and found that cholinergic transmission is more transient than GABAergic transmission, attributable to differential postsynaptic receptor kinetics. Furthermore, in the rabbit retina, GABAergic inputs to DSGCs saturates at a lower contrast than cholinergic and glutamatergic inputs do, indicating that differential gain control system contributes to SAC direction selectivity (34). Also, in the mouse retina, spatiotemporal properties of cholinergic and GABAergic transmissions are distinct in response to low-contrast visual stimuli (28). These reports revealed that differential mechanisms govern the dual synaptic transmissions from SACs to RGCs, although both acetylcholine and GABA are released from single SACs.

Previous reports have shown that preferred and null directional moving stimuli evoke steeper or shallower EPSPs in SACs, respectively (24, 26, 27). Our results show that EPSCs (cholinergic) and IPSCs (GABAergic) are differentially regulated by presynaptic depolarization kinetics. The differential effects might be shaped by presynaptic release dynamics as well as postsynaptic receptor properties, including receptor kinetics and pre-postsynaptic locations, either point-to-point synaptic or volume transmission. Corresponding to SAC release of GABA and acetylcholine, postsynaptic receptors in RGCs are primarily nicotinic acetylcholine and GABA-A receptors. Both receptors rapidly desensitize, which would not explain the distinct temporal sensitivities between EPSCs and IPSCs.

However, differences in the site of presynaptic transmitter release and postsynaptic synaptic locations might affect the temporal sensitivity. In SAC-DSGC synaptic transmission, GABA transmission is synaptic, whereas cholinergic is considered to occur by volume transmission (29, 35). Bolus synaptic release by sharp depolarization would activate both types of receptors. However, sustained transmitter release might affect the volume transmission by diffusion or transporter activities before transmitters reach the distant postsynaptic site. Therefore, our results might be explained by different cholinergic and GABAergic release dynamics such as distinct Ca^2+^ sensitivities (31) and presynaptic-postsynaptic location differences between GABAergic and cholinergic transmission.

Furthermore, Pottackal et al. (29) observed a difference in latency between GABAergic and cholinergic transmissions from SACs, and concluded that cholinergic transmission is paracrine in nature. However, we did not see a difference in latency between the stimulus and response onset for EPSCs and IPSCs. This is probably due to our experimental limitation that our data consisted of a diverse group of ganglion cell types. This heterogenous population may consist of cells that receive SAC inputs by point-to-point and paracrine transmission.

How does the temporal sensitivity difference between EPSCs and IPSCs affect the direction selectivity in ganglion cells? Our results indicate that rapid depolarization of SAC dendrites induces greater EPSCs in postsynaptic ganglion cells than slower, more prolonged SAC depolarization, whereas GABAergic inhibition was less sensitive to depolarization kinetics (**Figure 2B**, right). These results indicate that null-directional motion input to SACs generates slower depolarization, leading to a net balance toward inhibition of RGCs. This EPSC/IPSC balance may contribute to the transmission of directional information from SACs to postsynaptic ganglion cells.

As RGCs were blindly patched in this study, recordings of postsynaptic cells contained a variety of RGC types, including ON, OFF, and ON-OFF RGCs, most likely including DSGCs in addition to other types. Since SACs are classically known for their central role in communicating motion information laterally across retinal circuits, our data suggest that SACs could also signal motion information to other RGC types as well. Although the significance of SAC transmission to non-DS RGCs is uncertain, all RGCs we included exhibited EPSCs, IPSCs, or both during optogenetic stimuli.

Finally, SACs’ compartmentalization and gating system (25, 36) isolates the soma from the rest of the dendritic field, and patch clamp recordings from the soma may not fully detect the electric changes in dendrites. However, our results indicate that subtle changes in SAC depolarization as revealed at the soma might contribute to the SAC’s directionally selective transmitter release.

## Data availability statement

The original contributions presented in the study are included in the article/supplementary material. Further inquiries can be directed to the corresponding author.

## Ethics statement

The animal study was approved by Institutional Animal Care and Use Committee, Wayne State University. The study was conducted in accordance with the local legislation and institutional requirements.

## Author contributions

TI, CH, & JB conducted experiments, JB & CH analyzed data, TI wrote the manuscript, TI, CH, & JB edited the manuscript and agreed the final version.
